# Investigation into the mechanical properties and impact tendency of coal-rock composite structures with peridynamics: A study on predicting the occurrence tendency of dynamic pressure in coal-rock structures

**DOI:** 10.1371/journal.pone.0314927

**Published:** 2024-12-04

**Authors:** Jinqiu Dai, Mingchao Zhao, Zhenkang Wang, Han Gao

**Affiliations:** 1 School of Civil and Architectural Engineering, Anyang Institute of Technology, Anyang, China; 2 China Coal Technology and Engineering Group Shenyang Design & Research Institute, Shenyang, China; China University of Mining and Technology, CHINA

## Abstract

Due to the difficulty in predicting the occurrence of rockburst in deep mining areas,this paper proposes that the use of Peridynamics to analyze the mechanical characteristics of coal-rock composite structures under loading conditions from the perspective of energy, Determine the impact tendency of coal rock composite structures by combining rock elastic deformation energy index (*W*ET) and rock impact energy index (*W*CF); Use Lammps software to simulate the loading of composite structural materials and compare and verify with experimental results, to more accurately determine the tendency of rockburst occurrence in different coal-rock composite structures.The results indicate that after the specimen reaches the yield stress, the sample exhibits an "X" shaped failure. The coal body has a significant impact on the overall model’s fragmentation, and different combination states and ratios can affect the degree of damage in the coal-rock composite structure. which has important theoretical value for rock mechanics research. The research results can reduce the occurrence of rockburst accidents, the difficulty of mine support, and the cost of mining engineering, as well as improve mine safety levels.

## Introduction

As shallow energy resources in China are gradually depleted, the depth and breadth of energy exploitation increase, resulting in an alarming increase in rockbursts. Such incidents often cause rapid and unexpected falls or ejection of coal or rocks, posing a serious threat to the safety of subterranean workers, equipment, and materials. Moreover, the collapse of roadway support in the pre-mining area and the occurrence of disasters such as earthquakes are also attributed to the serious nature of rockbursts [1,2]. To prevent rockbursts and ensure safety in underground coal reservoirs, it is crucial to evaluate their likelihood.

The phenomenon of rockburst arises from the sudden discharge of a significant energy reserve that has built up within the coal mass, responding to either mine or constraining pressures, featuring traits of both seamless and indefinite natures [3,4]. A considerable variety of sandstone-related rockburst accidents were analyzed by Duan et al [2], who proposed a range of dissipation approaches to mitigate these incidents, anchored in the theory of dynamic and static charge superposition. Wang [5] examined the acoustic emission response features of a coal body during various phases such as the primary crack closure stage, linear elastic stage, elastic-plastic deformation stage, and strain softening stage, under loading conditions. He also developed a ground sound-based monitoring and early warning model to assess rockburst risk levels. Nevertheless, coal bodies in underground spaces do not exist independently; many coal-rock combinations are interlayered to form composite structures. These composite structures differ significantly in structural characteristics, compositional ratios, sequence, and even load-bearing surface properties [6,7]. Studying the internal energy changes under loading conditions for only a single rock or coal body is incomplete and cannot fully reflect the actual working conditions of the mining area. Therefore, analyzing the mechanical properties of coal-rock composite structures is particularly important.

Researchers in both domestic and international settings have extensively studied the mechanical properties of coal-rock composite structures, as well as the failure processes and internal energy evolution involved. These investigations have primarily been conducted through indoor simulations of both uniaxial and triaxial compression experiments, in addition to a significant number of numerical simulations. As for specific research, Li et al [8,9] utilized indoor impact experiments to examine the coal-rock composite structure. The energy dissipation and crushing characteristics of the coal-rock composite structure under load conditions were analyzed, and the rules governing crack propagation and derivation were obtained by employing the fractal dimension. Liu et al [10] developed a constitutive model of the coal-rock composite structure based on the two models of series damage body and Newtonian body and determined the influence of rock mass on the mechanical properties of coal within the structure. Zuo [11–13] investigated the mechanical properties and post-peak failure characteristics of the coal-rock composite structure under load conditions and summarised the law of crack propagation in the coal-rock composite structure after yield. Zhao [14] found that the rock burst of the coal-rock combination structure occurs due to microscopic and disorderly competition within the coal-rock system, as observed from a microscopic standpoint during the loading process. Consequently, there has been an increased focus on studying the energy perspective of the failure law during rock burst occurrences with the increase in mining depth. However, during the calculation of energy analysis, the differential equations that are typically employed often yield discontinuous solutions and do not possess a partial derivative of position, resulting in analysis difficulties and inaccuracies in the results [15,16].

Peridynamics (PD theory) is a non-local modeling theory that describes the mechanical properties of brittle materials by integrating equations of motion [17]. Furthermore, PD theory presents various analytical methods, including bond-based PD theory. This method effectively resolves the aforementioned issues and has exhibited high accuracy in both macro and micro scales [18]. Based on previous research, this study conducts uniaxial compression experiments on coal-rock composite structures with different combination states, ratios, and load-bearing surfaces. It measures the mechanical parameters of the varied coal-rock composite structures. The impact propensity of varied coal-rock composite formations is assessed through a combination of two energy indexes-rock elastic deformation energy index (*W*_ET_) and traditional rock impact energy index (*W*_CF_). The coal-rock composite structure was loaded through the Peridynamics and its internal energy changes were analyzed via the Lammps software to verify the experiment. This simulation provides an effective and accurate prediction for rock burst issues.

## Impact experiment of coal-rock composite structure sample preparation

**Fig 1:**
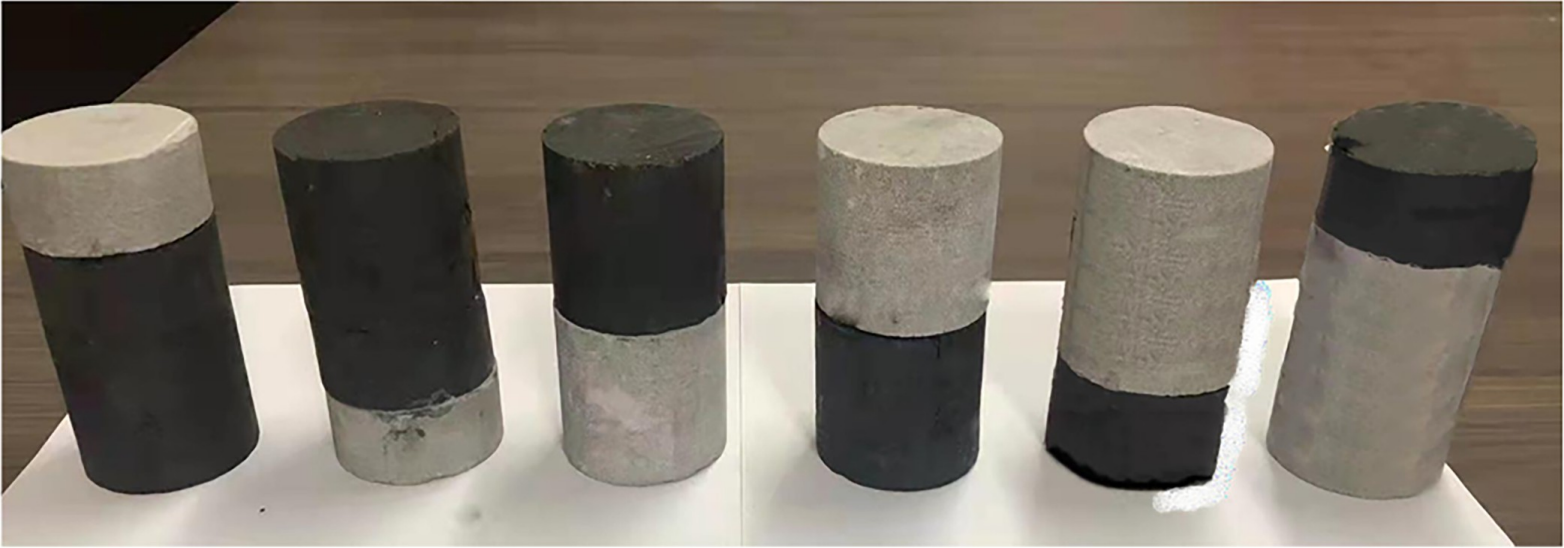
Actual item diagram of some coal-rock composite structures.

The coal and rock samples from the long flame coal seam in Fuxin Basin, Liaoning Province, have been selected. The non-parallelism of the processed end faces meets the requirements of GB/T23561.7–2009, the national standard for the mechanical properties of coal-rock composite structures, and coal-rock single samples [19–21]. Since the coal-rock combination ratio in engineering cases where dynamic disasters occur is mostly 1:1 or 1:2, the coal and rock are cut to different heights accordingly, including 100mm, 66mm, 50mm, 34mm, and so on, to create *Φ*50mm×100mm cylindrical sample of coal-rock composite structures. Different combination states and ratios were employed for this purpose, with an error margin of less than 0.2mm. After bonding the samples with AB adhesive, hey were allowed to stand for 24 hours to solidify. The methodology was adopted from reference [22]. As presented in Fig 1, the physical diagram depicts samples of differing coal-rock combination structures where M symbolizes coal rock and S represents sandstone. Table 1 displays the sample size numbers. For instance, MS denotes the structure of a coal-rock combination consisting of upper coal and lower rock.

**Table 1:** Different sizes of coal-rock composite structures.

Composite type	Number	Dimension/mm	Dimension scale
**A unitary structure**	M	100	1
	S	100	1
**Binary composite structure**	MS-1	34:66	1:2
	MS-2	50:50	1:1
	MS-3	66:34	2:1
	SM-1	34:66	1:2
	SM-2	50:50	1:1
	SM-3	66:34	2:1

### Experimental method

**Fig 2:**
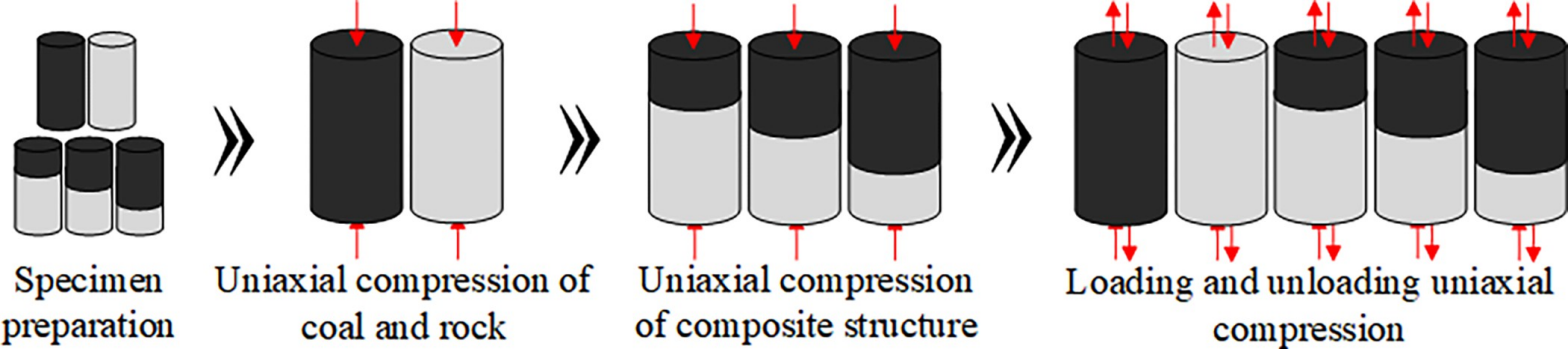
Experimental flowchart.

During the actual impact ground pressure process, the energy accumulated within the coal-rock composite structure causes both coal and rock to fail simultaneously. Therefore, the ratio of the compressive strengths of coal (M) and sandstone (S) is used to obtain the length ratio of coal to sandstone, ensuring that both coal and rock reach their compressive limits simultaneously during the experiment. The prepared coal-rock composite specimens were divided into two groups. Using a universal testing machine, a uniaxial compression test was first conducted on one group, with full stress-strain curves plotted for different specimens. Based on these curves, the impact energy (*c*) and compressive strength of each specimen were calculated. Then, a similar uniaxial compression test was performed on the second group according to the compressive strengths of each specimen. Once the stress reached 80% to 90% of each specimen’s compressive strength, unloading was conducted to allow stress-strain data to rebound, and the elastic energy (*t*) was calculated from the rebound curve. The loading rate for all experiments was set at 0.002 mm/s, and each experiment was repeated five times. The average value was taken as the experimental result. The experimental process is shown in Fig 2.

## Experiment results

**Fig 3:**
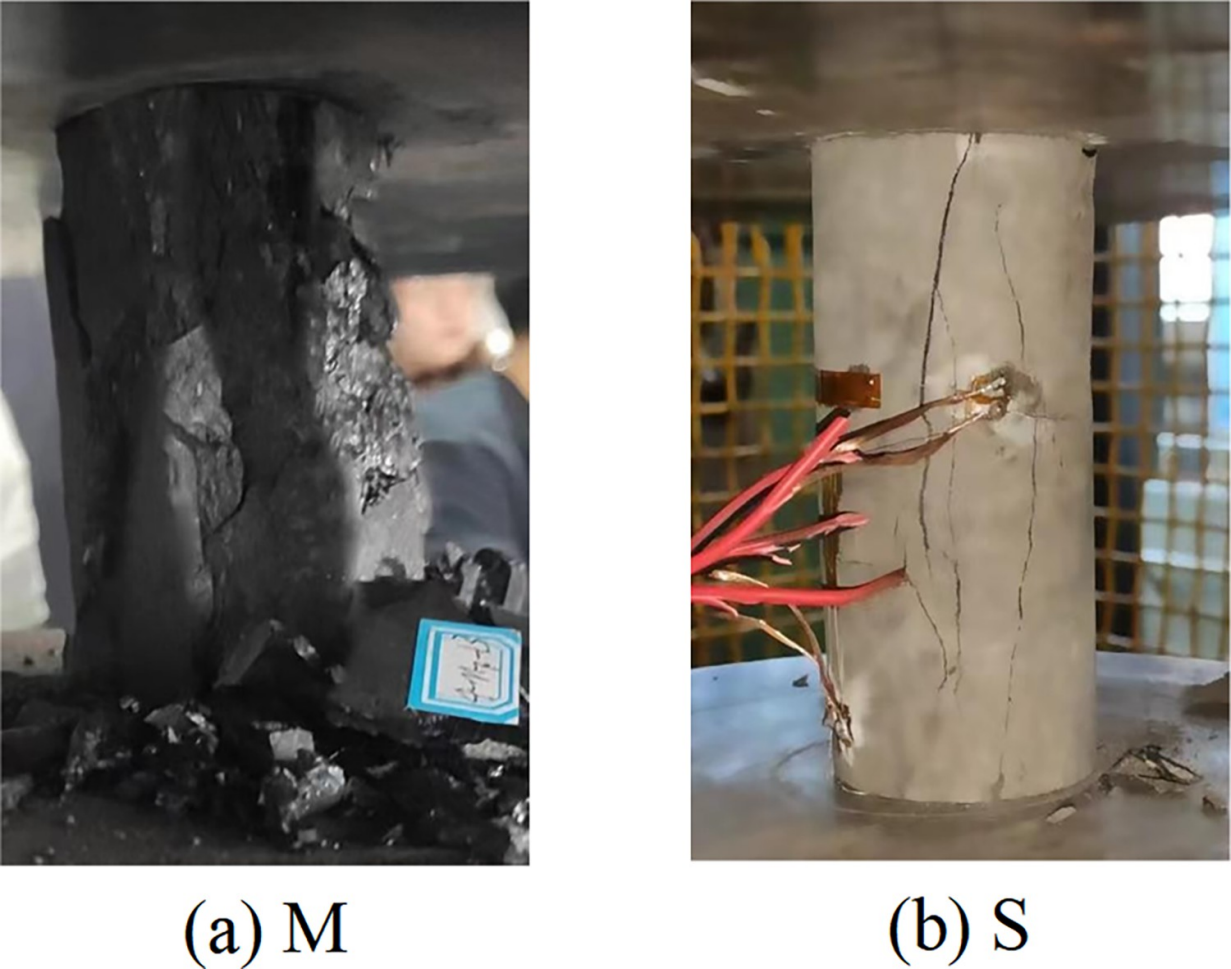
Failure morphology of coal and rock.

**Fig 4:**
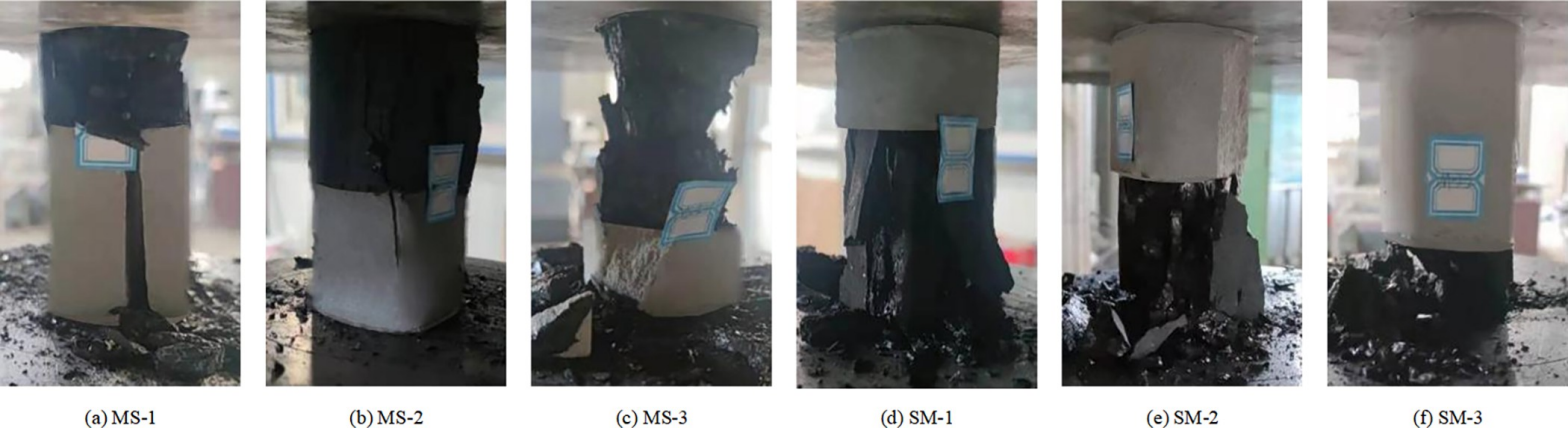
Failure morphology of coal-rock composite structure specimens.

Uniaxial compression experiments were conducted on eight groups of coal-rock monomers and coal-rock composite structures according to the experimental scheme. The failure modes of various specimens after loading are depicted in Figs 3 and 4, whereas the mechanical parameter data of different samples are presented in Tables 2 and 3.

**Table 2:** Mechanical parameters of coal and rock monomer.

Number	*σ* /MPa	*t* /(kJ∙m^-3^)	*c* /(kJ∙m^-3^)
**M**	15.16	0.104	0.021
**S**	99.75	15.365	8.265

**Table 3:** Mechanical parameters of coal-rock composite structure specimens.

Number	*σ* /MPa	*t* /(kJ∙m^-3^)	*c* /(kJ∙m^-3^)
**MS-1**	32.05	0.609	2.498
**MS-2**	19.25	0.299	0.125
**MS-3**	17.06	0.201	0.067
**SM-1**	16.73	0.230	0.079
**SM-2**	18.62	0.310	0.132
**SM-3**	30.19	0.917	3.875

### Compressive strength analysis of coal-rock composite structure

**Fig 5:**
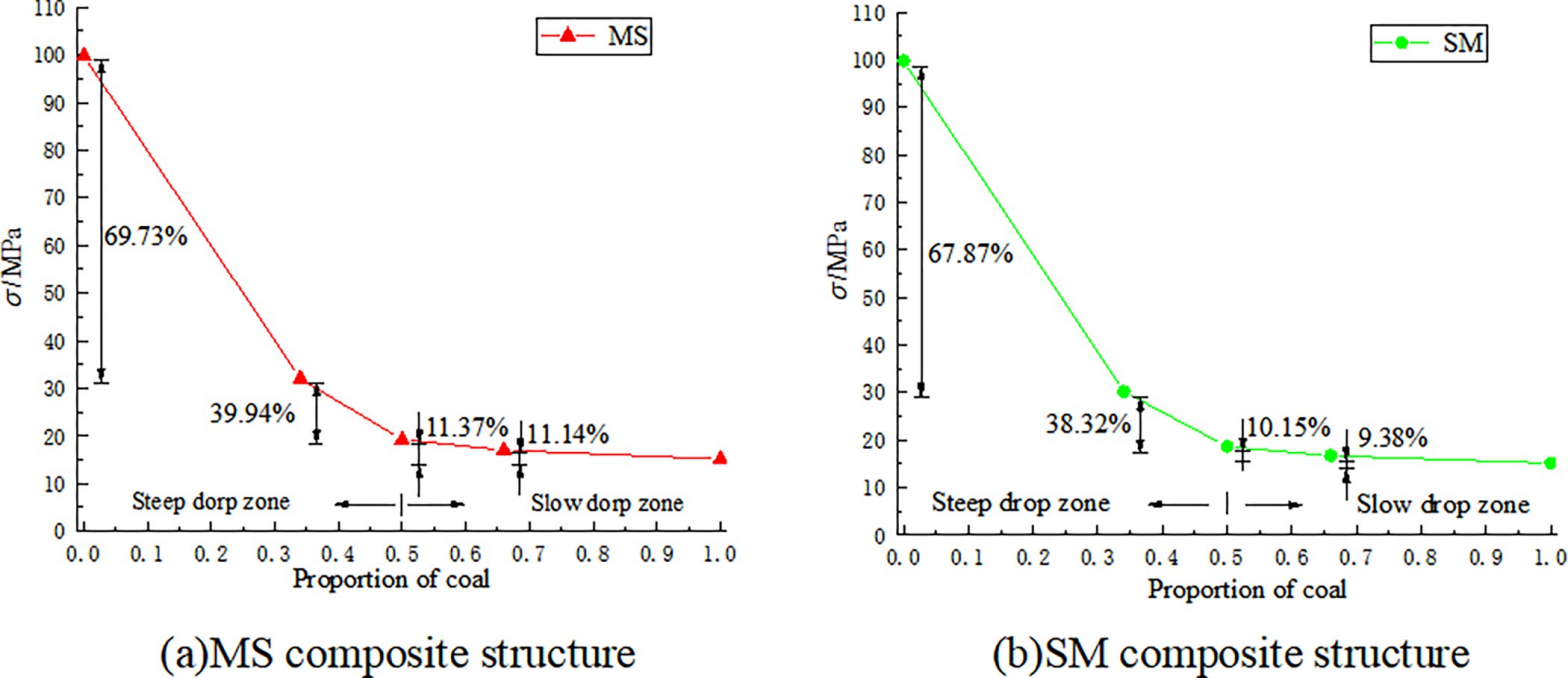
The relationship between the compressive strength of coal-rock composite structures, coal monomers, and rock monomers and the proportion of coal.

The compressive strength of different coal-rock composite structures was between the compressive strength of coal monomers and rock monomers, and they were more inclined towards weak components, that is, the proportion of coal. Thus, the compressive strength of a coal-rock composite is highly influenced by the proportion of weaker components in the composite structure. The correlation between the compressive strength of varying coal-rock composite structures, coal monomers, rock monomers, and coal proportion is illustrated in Fig 5.

As shown by Fig 5, When the coal-rock composite structure contains coal, the compressive strength significantly decreases, with MS composite structure decreasing by 67.87% and SM composite structure decreasing by 69.73%, indicating that the upper rock and lower coal composite have stronger compressive resistance; The compressive strength of composite structure is related to the proportion of coal. With the increase of the proportion of coal in the composite structure, the compressive strength of composite structure gradually decreases. When the proportion of coal is less than 50%, the compressive strength decreases significantly. When the proportion of coal is more than 50%, the reduction is slow. MS-3 and SM-1 are the two composite structure with the lowest compressive strength among the corresponding coal-rock composite structure, which are 16.73 MPa and 17.06 MPa, respectively, which are only about 10% higher than the coal monomer sample.

This is basically consistent with the macroscopic failure effect of different composite structure shown in Fig 4, that is, after the MS composite structure is loaded, the coal and the rock mass are broken macroscopically, while after the SM composite structure is loaded, the coal is broken partially, and the overall structure of the upper rock mass is relatively complete.

### Comparative analysis of elastic energy of coal-rock composite structure

According to the data on the elastic energy (*e*) of composite structure, the elastic energy of composite structure with coal participation is smaller than that of rock masses. This is because the limit energy storage of coal is small, while the limit energy storage of rock is high. During the loading process of the composite structure, the elastic energy stored in the coal reaches the limit, and the combined structure begins to produce macro damage. Therefore, the elastic energy of the coal-rock composite structure depends on the limit energy storage of the weak components.

**Fig 6:**
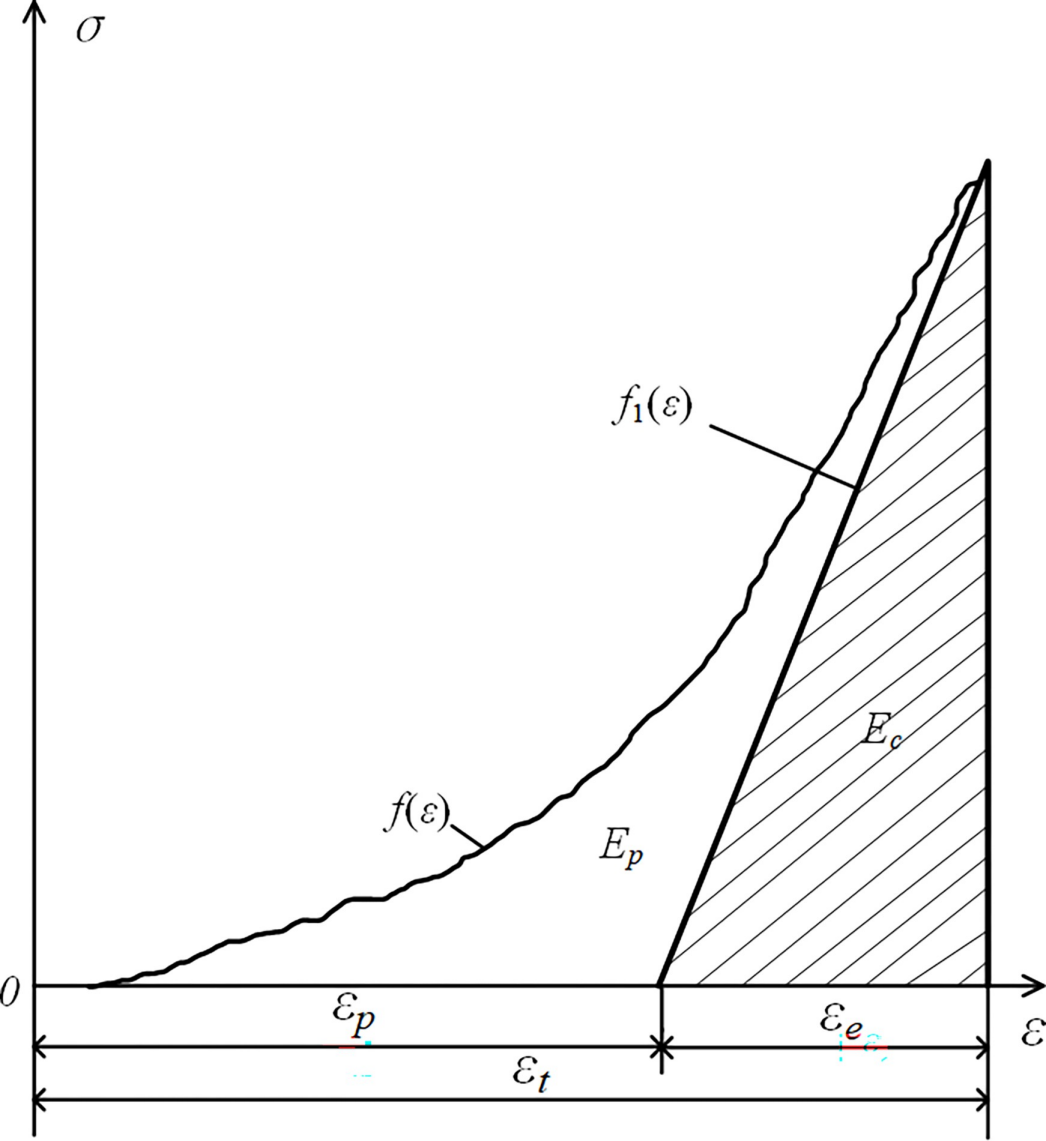
Elastic energy (*e*) calculation *W*_ET_ model diagram (where *f* (*ε*) is the loading curve; *f*_1_(*ε*) is the unloading curve).

In 1981, Polish scholar Kidybinski innovatively proposed the ratio of elastic strain energy to plastic strain energy (Elastic Deformation Energy Index, *W*_ET_) as an indicator for assessing the tendency of impact ground pressure [23]. This is primarily calculated using the area enclosed by the loading and unloading curves, as illustrated in Fig 6. The calculation method is shown in Eq (1).


$$W_{ET}=\frac{E_{e}}{E_{p}}$$ (1)

Where *E*_*e*_ is elastic strain energy; *E*_*p*_ is plastic energy.

The "Classification of Coal Seam Impact Tendency and Determination Method of Index" (MT/T174-2000) provides the criteria for determining the occurrence of impact ground pressure in coal and rock using *W*_ET_ [24], as shown in Eqs (2) and (3).


$$\left\{ \begin{array}{l} W_{\mathrm{ET}}\geq 5,\mathrm{strength}\\ 2\leq W_{\mathrm{ET}}<5,\mathrm{feebleness}\\ W_{\mathrm{ET}}<2,\mathrm{nil} \end{array}\right.$$ (2)

$$\left\{ \begin{array}{l} W_{\mathrm{ET}}\geq 15,\mathrm{strength}\\ 10\leq W_{\mathrm{ET}}<15,\mathrm{feebleness}\\ W_{\mathrm{ET}}<10,\mathrm{nil} \end{array}\right.$$ (3)

Based on Eqs (2) and (3), the criteria for determining the impact tendency of coal-rock composite structures using *W*_ET_ can be derived, as shown in Eq (4).


$$\left\{ \begin{array}{l} W_{\mathrm{ET}}\geq 10,\mathrm{strength}\\ 7\leq W_{\mathrm{ET}}<10,\mathrm{feebleness}\\ W_{\mathrm{ET}}<7,\mathrm{nil} \end{array}\right.$$ (4)

**Fig 7:**
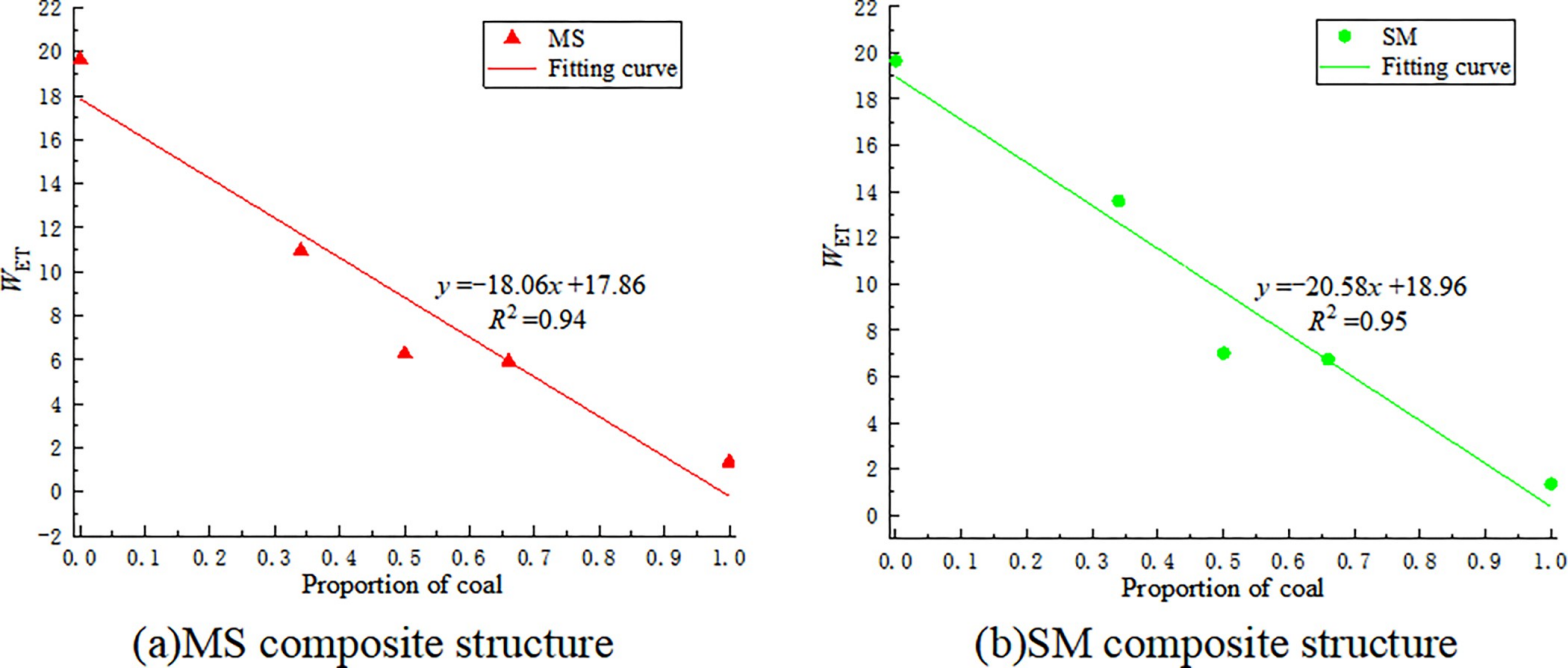
The relationship between the *W*_ET_ value of coal-rock composite structure, coal monomer.

Based on the experimental data in Tables 2 and 3, the *W*_ET_ for coal-rock monomers and different coal-rock composite structures is calculated, as shown in Table 4. The relationship between the *W*_ET_ values of different coal-rock composite structures and the proportion of coal is shown in Fig 7.

**Table 4:** *W*_ET_ values of coal-rock composite structure, coal monomer and rock monomer.

Name	M	S	MS-1	MS-2	MS-3	SM-1	SM-2	SM-3
***W***_**ET**_	1.35	19.65	10.95	6.26	5.93	6.74	7.01	13.59

According to Fig 7, the *W*_ET_ values of different coal-rock composite structures show a linear decreasing trend with the increase in the proportion of coal. Based on the criteria in Eqs (2)–(4), the impact tendency of coal-rock composite structures should lie between the impact tendencies of the individual coal and rock monomers. It is roughly determined that the structures S, MS-1, and SM-3 have a strong impact tendency, while the remaining structures have a weak or no impact tendency. Among the coal-rock composite structures, the structure with a coal-to-rock ratio of 1:2 has the strongest impact tendency, with the highest *W*_ET_ value for SM-3. This is because the load-bearing surface is relatively harder sandstone, and sandstone has greater stiffness, making it more difficult to fracture. In coal-rock composite structures, it is easier to accumulate a large amount of elastic energy, increasing the likelihood of impact ground pressure. In actual engineering practice, impact ground pressure accidents are more likely to occur at the roof position.

### Comparative analysis of impact energy of coal-rock composite structure

According to the data on impact energy (*c*) of the composite structures, the impact energy generated by coal-involved composite structures is smaller than that of rock-only sections. This is because the elastic energy stored in the coal body is lower, and when the coal’s storage capacity reaches its limit, the resulting impact damage is smaller than that of the rock. Therefore, the impact energy generated by different coal-rock composite structures depends on the ultimate energy storage capacity of the weaker component.

**Fig 8:**
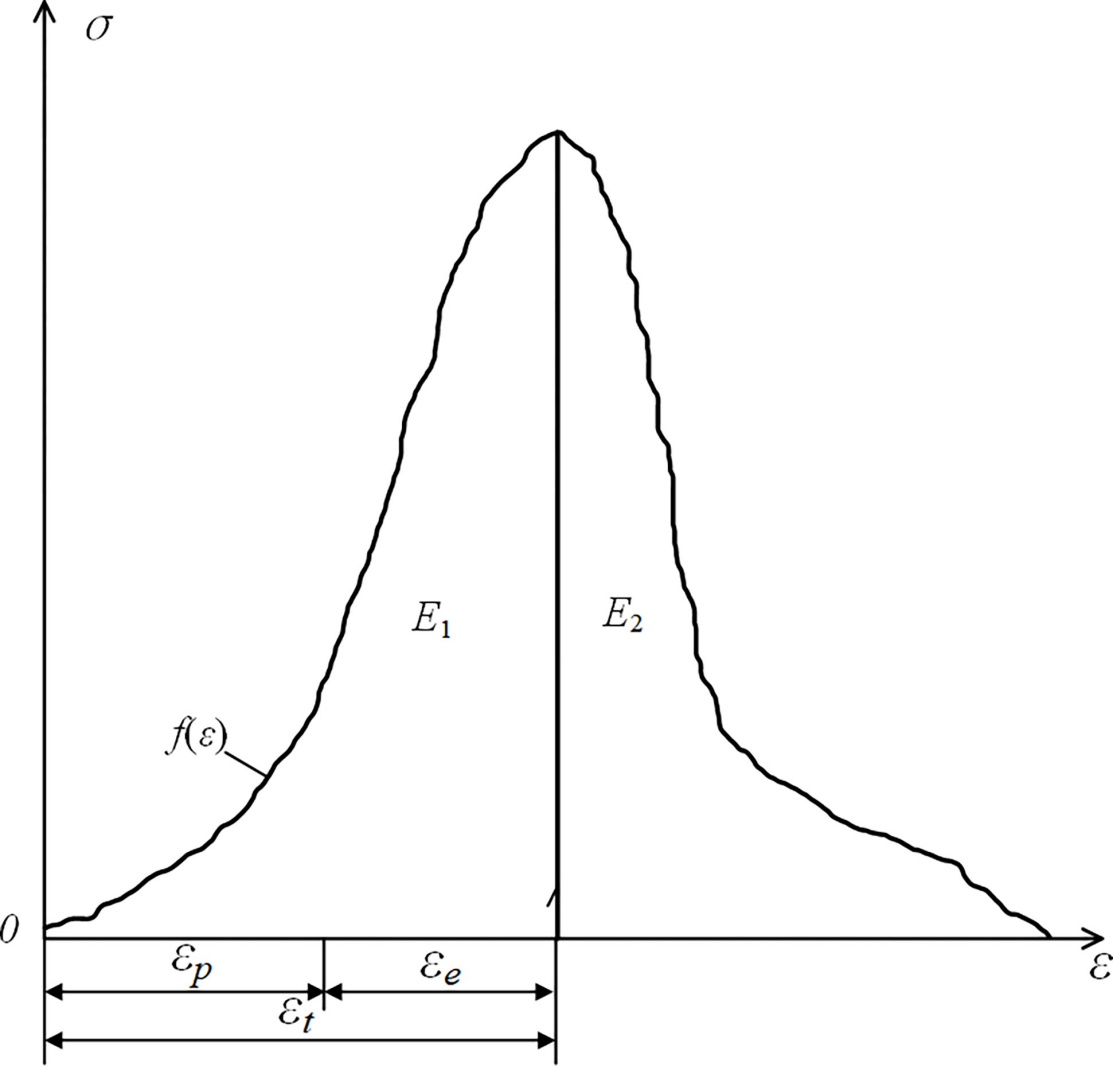
Impact energy (*c*) calculation *W*_CF_ model diagram.

Since using only the *W*_ET_ value to determine the impact tendency of coal-rock monomers and different coal-rock composite structures may lead to errors, the Rock Impact Energy Index (*W*_CF_), which is the ratio of the area enclosed before the peak (*E*_1_) to the area enclosed after the peak (*E*_2_), is used to reassess the impact tendency of each sample. The principle is shown in Fig 8, and the calculation method is shown in Eq (5).


$$W_{\mathrm{CF}}=\frac{E_{1}}{E_{2}}$$ (5)

The criteria are as follows:

$$\left\{ \begin{array}{l} W_{\mathrm{CF}}\geq 3,\mathrm{strength}\\ 2\leq W_{\mathrm{CF}}<3,\mathrm{feebleness}\\ W_{\mathrm{CF}}<2,\mathrm{nil} \end{array}\right.$$ (6)

**Fig 9:**
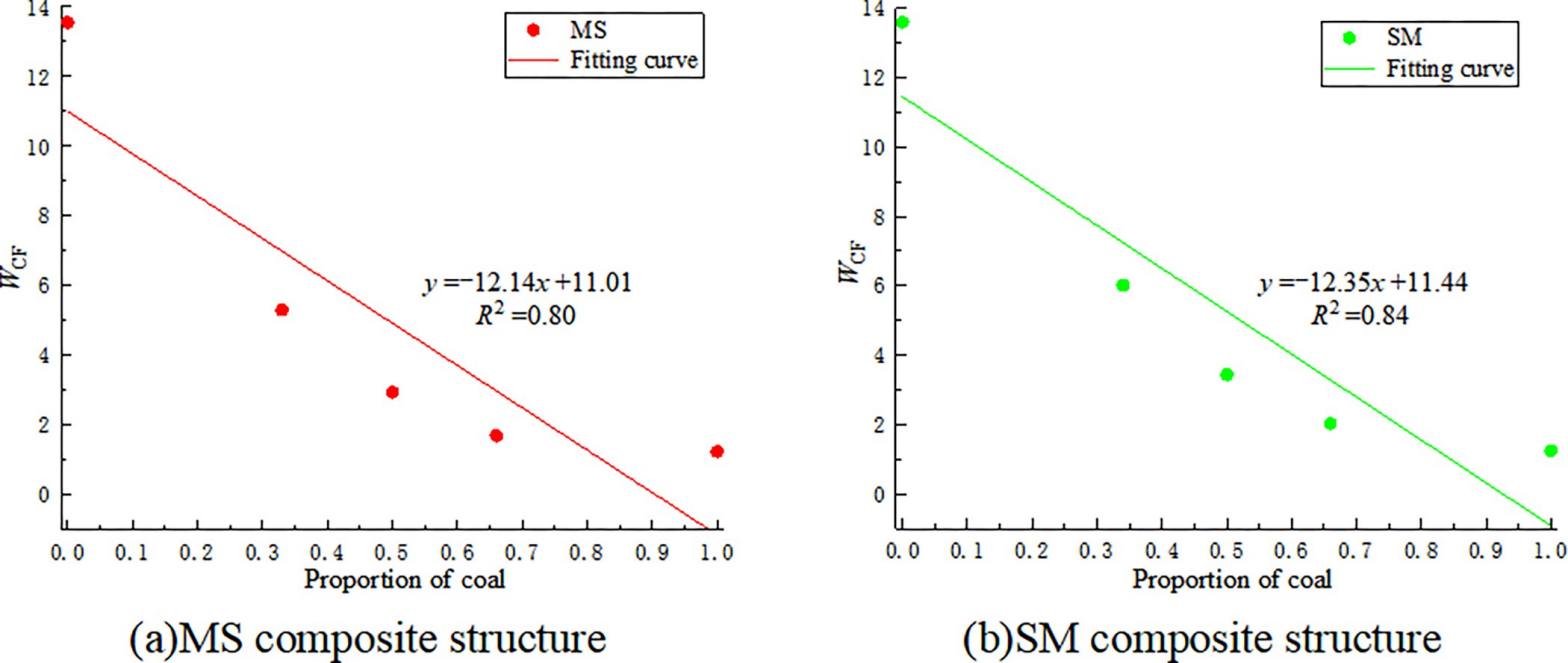
The relationship between the *W*_CF_ value of coal-rock composite structure, coal monomer.

Based on the experimental data in Tables 2 and 3, the Rock Impact Energy Index (*W*_CF_) for coal-rock monomers and different coal-rock composite structures is calculated, as shown in Table 5. The relationship between the *W*_CF_ values of different coal-rock composite structures and the proportion of coal is shown in Fig 9.

**Table 5:** *W*_CF_ values of coal-rock composite structure, coal monomer and rock monomer.

Name	M	S	MS-1	MS-2	MS-3	SM-1	SM-2	SM-3
***W***_**CF**_	1.25	13.55	5.31	2.95	1.71	2.03	3.44	6.02

According to Fig 9, the *W*_CF_ values of different coal-rock composite structures also show a linear decreasing trend with the increase in the proportion of coal. Based on Eq (6), the impact tendency of coal-rock composite structures should lie between the impact tendencies of the individual coal and rock monomers. It is determined that S, MS-1, SM-2, and SM-3 have a strong impact tendency, while the remaining structures have a weak or no impact tendency. This is roughly the same as the impact tendency determined by the *W*_ET_. Among the coal-rock composite structures, the structure with a coal-to-rock ratio of 1:2 has the strongest impact tendency, with SM-3 having the highest *W*_CF_ value. This is because, during loading, this composite structure stores the maximum elastic energy. As the working face advances, the accumulated energy reaches the storage limit, releasing a large amount of energy, leading to more severe and destructive impact ground pressure. Therefore, in actual engineering practice, treatments such as cutting seams and drilling holes in the roof are done to release the energy accumulated in the roof and reduce its energy storage limit, thereby effectively preventing the occurrence of impact ground pressure. In comparison, impact ground pressure phenomena are relatively less frequent in structures with coal over rock (coal on top, rock on the bottom) than in structures with rock over coal (rock on top, coal on the bottom).

## Establishment of mechanical model of coal-rock composite structure

**Fig 10:**
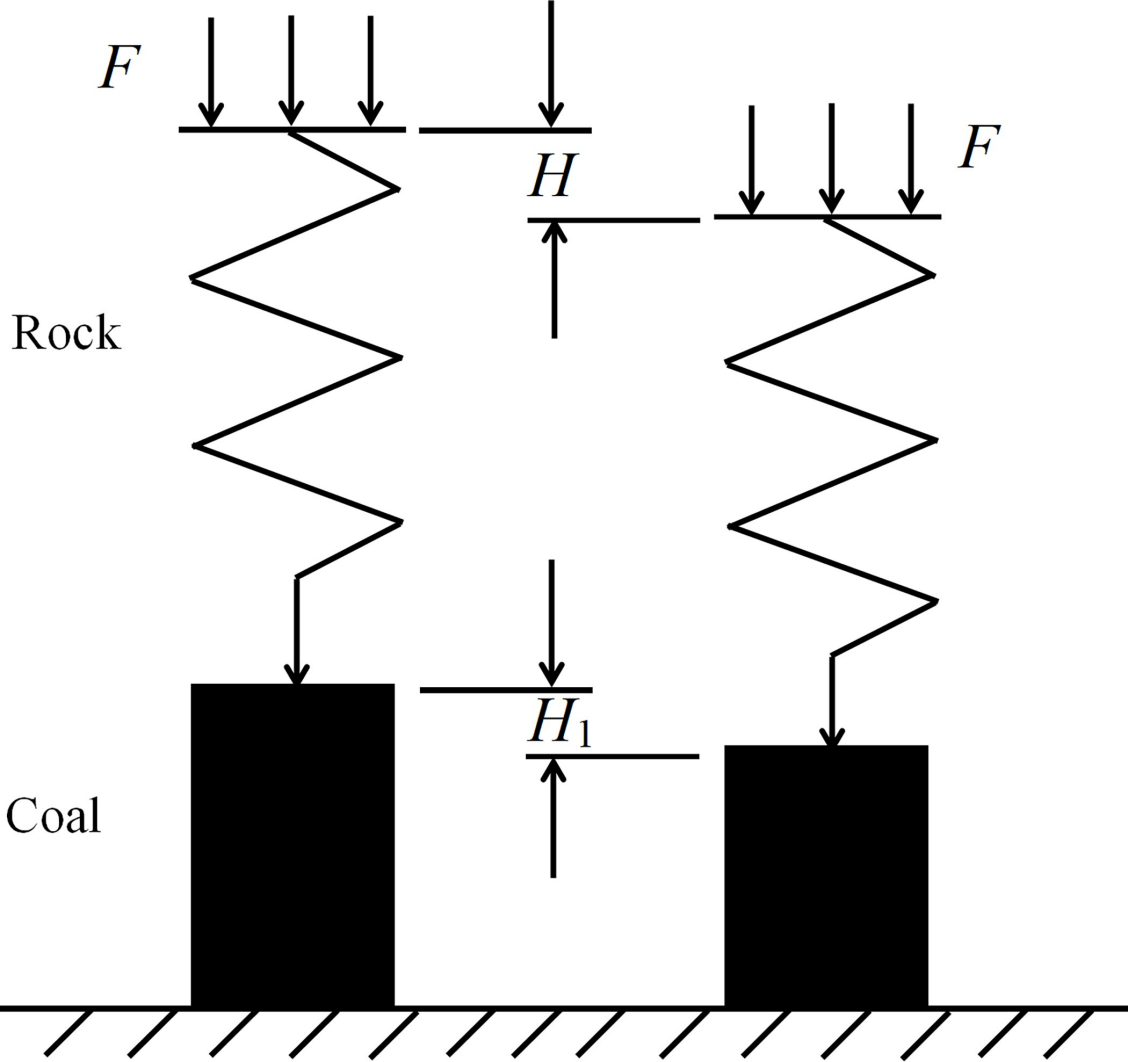
Mechanical model diagram of upper rock and lower coal combination structure.

During the process of coal-rock composite structure instability and failure under load, the coal component initially reaches its compressive strength limit. Subsequently, the rock mass can be viewed as being in a linear elastic deformation stage and is simplified as a spring structure that closely adheres to the viscoelastic material Maxwell model [25,26]. This means that the spring element is correlated with the damping element in a series. Thus, we have formulated a mechanical model of the composite structure consisting of upper rock and lower coal subjected to load, as illustrated in Fig 10. The model comprises *F*, the uniform load of the composite structure, *H*, the total deformation of the composite structure, and *H*_1_, the deformation of the coal body.

When a uniformly distributed load *F* is applied to the rock mass, the force *F*_1_ acts on the rock mass and the force *F*_2_ acts on the coal body.

$$\left\{ \begin{array}{l} F_{1}=M_{1}\frac{d^{2}H}{dt^{2}}+k(H-H_{1})\\ F_{2}=f(H_{1},t) \end{array}\right.$$ (7)

where *M*_1_ is the quality of roof strata; *k* is the stiffness of the roof strata.

When the combination’s structure reaches equilibrium.


$$F_{1}=F_{2}=F$$ (8)

Because the force in the coal-rock composite structure is dependent on displacement and time, the combined structure experiences zero acceleration:

$$\frac{d^{2}H}{dt^{2}}=0$$ (9)

At this time, the increase in forces *F*_1_ and *F*_2_ can be observed:

$$\left\{ \begin{array}{l} \Delta F_{1}=-k\Delta H_{1}\\ \Delta F_{2}=f^{\prime }(H_{1},t)\cdot \Delta H_{1}=\frac{df(H_{1},t)}{dH_{1}}\cdot \Delta H_{1} \end{array}\right.$$ (10)

Then, the energy change of the single part of coal and rock is:

$$\left\{ \begin{array}{l} A_{1}=(F_{1}+\frac{1}{2}\Delta F_{1})\cdot \frac{\Delta H_{1}}{H}\\ A_{2}=(F_{2}+\frac{1}{2}\Delta F_{2})\cdot \frac{\Delta H_{1}}{H} \end{array}\right.$$ (11)

where *A*_1_ and *A*_2_ represent the amount of energy stored in the rock mass and the coal deposit, respectively

As the proportion of weak components in the coal-rock composite structure increases, the deformation *H*_1_ of the coal body also increases, resulting in the overall deformation *H* of the composite structure increasing. As a consequence, the energy *A*_2_ stored inside the coal body decreases gradually, subsequently leading to the reduction of energy storage in the composite structure. This supports the conclusion that the elastic and plastic energies decrease as the proportion of weaker components increases in the experimental outcomes.

According to Eq (11), the equilibrium relationship of the coal-rock composite structure can be determined as follows:

$$k+f^{\prime }(H_{1},t)\geq 0$$ (12)

Thus, the loading process of the coal-rock composite structure can be classified into three stages.

The first stage, known as the elastic stage:

$$\left\{ \begin{array}{l} k+f^{\prime }(H_{1},t)>0\\ \frac{df(H_{1},t)}{dH_{1}}=f^{\prime }(H_{1},t)>0\\ k>0 \end{array}\right.$$ (13)

At this stage, all components of the coal-rock composite structure are in the stage of elastic energy storage under the effect of a uniformly distributed load *F*. The stored elastic energy is gradually increasing, and the composite structure remains relatively stable.

The subsequent stage is the plastic stage:

$$\left\{ \begin{array}{l} k+f^{\prime }(H_{1},t)>0\\ \frac{df(H_{1},t)}{dH_{1}}=f^{\prime }(H_{1},t)<0\\ k>0 \end{array}\right.$$ (14)

At this stage, the individual coal particles undergo irreversible deformation caused by the significant strength of the overlying rock. The rock remains in the elastic stage, although some may experience slight deformation. The crushing of the lower coal body leads to the unloading of the overlying rock mass, thereby releasing elastic energy and accelerating the destruction of the coalrock combination.

The third stage is the residual strength stage:

$$\left\{ \begin{array}{l} k+f^{\prime }(H_{1},t)<0\\ \frac{df(H_{1},t)}{dH_{1}}=f^{\prime }(H_{1},t)<0\\ k>0 \end{array}\right.$$ (15)

At this stage, the coal-rock composite structure experiences brittle failure, resulting in a sudden change in strength. Additionally, impact failure occurs upon the release of a significant amount of energy.

**Fig 11:**
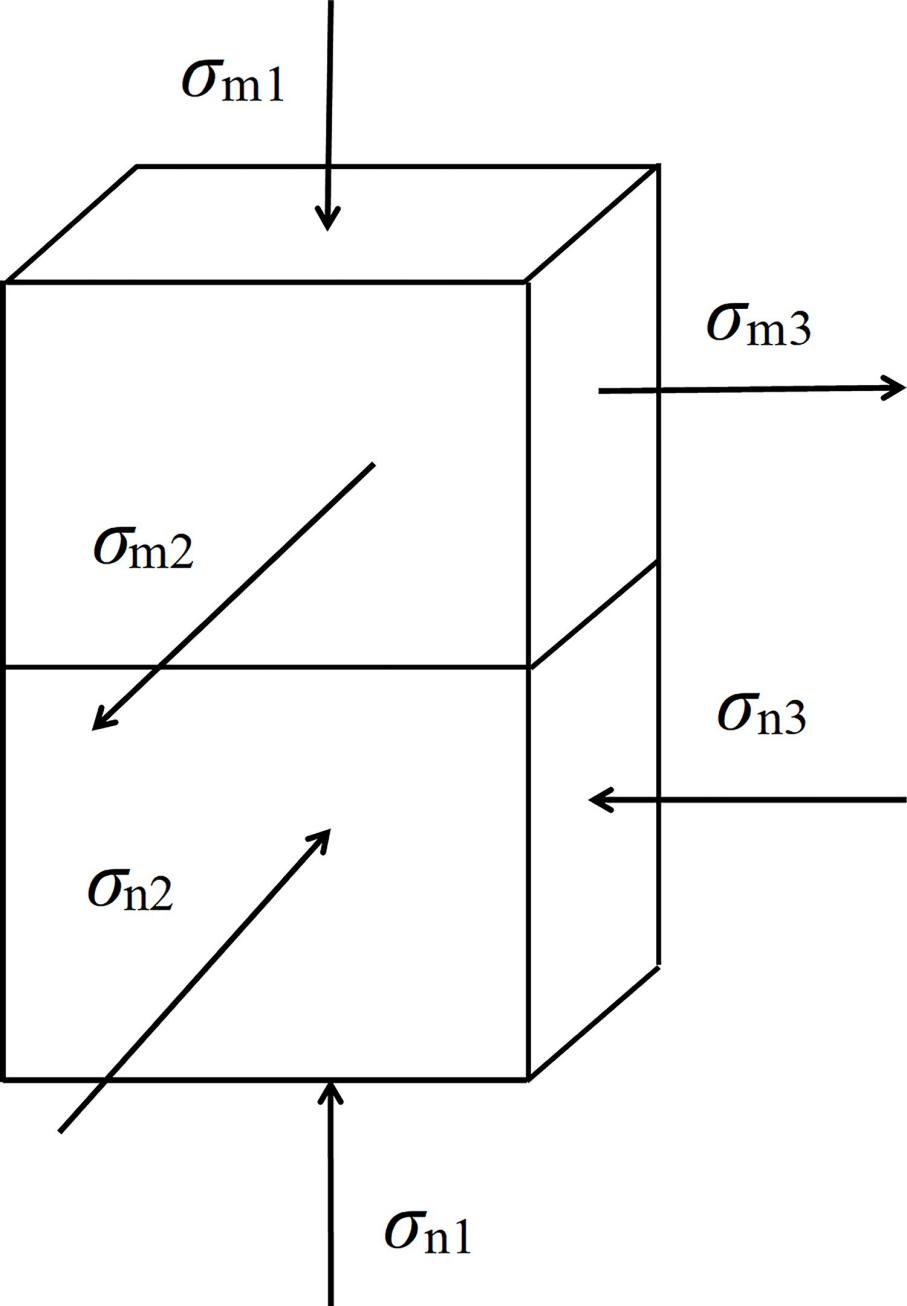
Stress diagram of the coal-rock composite structure interface (where *σ*_m1_ represents the axial stress of the rock mass; *σ*_m2_ and *σ*_m3_ denote the horizontal stress; *σ*_n1_ indicates the axial stress of the coal; *σ*_n2_ and *σ*_n3_ indicate the horizontal stress of the coal).

**Fig 12:**
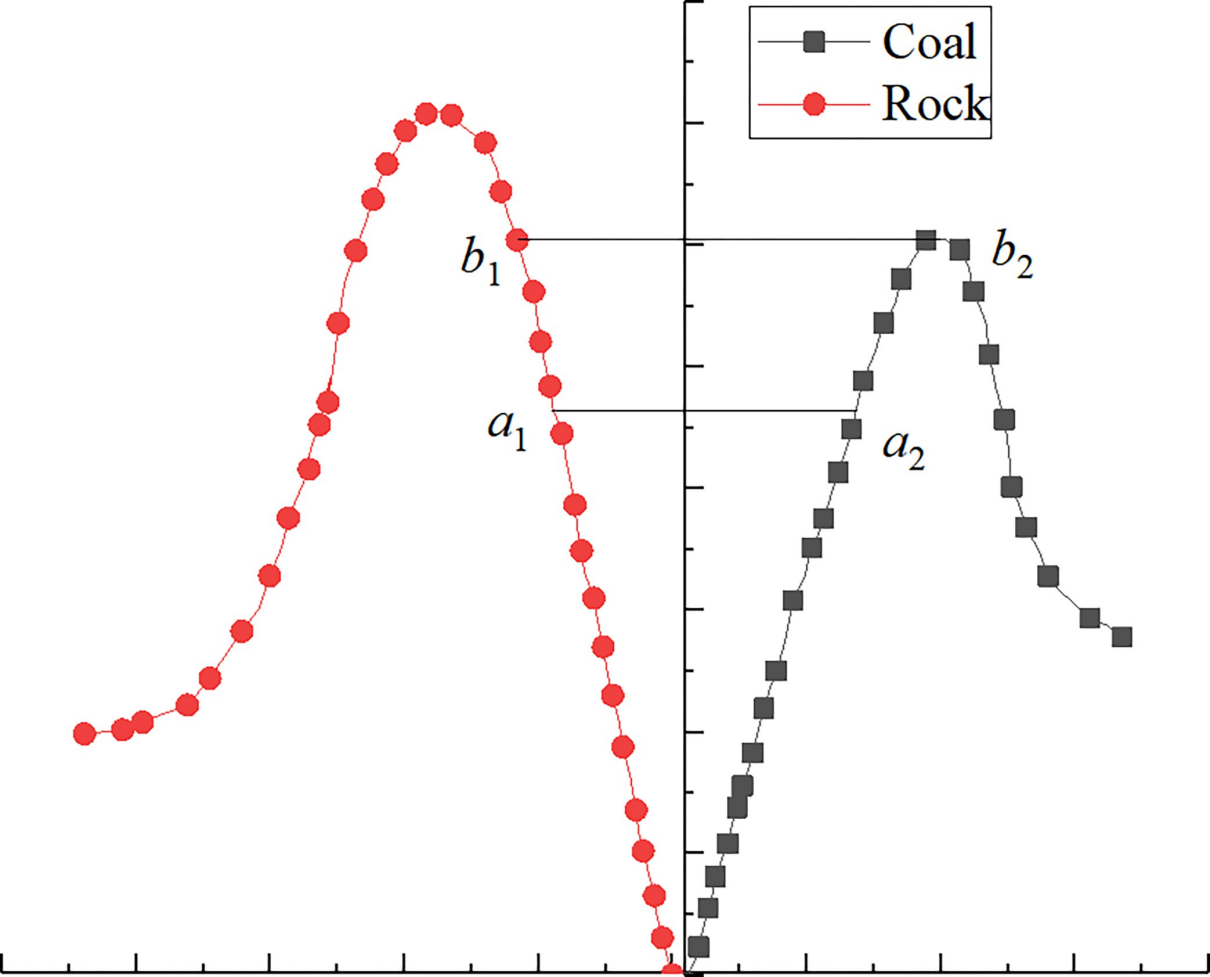
Mechanical change process of coal-rock composite structure.

The rock section of the composite structure experiences predominantly tensile stress during the loading process, whereas the coal part experiences mainly compressive stress. The stress at the interface of the composite structure is analyzed under the loading state, as indicated in Figs 11 and 12.

According to Figs 11 and 12, during the initial loading stage of diverse coal-rock institution compositions (known as the elastic stage), the rock mass and coal portions of the combination body interact, resulting in a continuous accumulation of elastic energy. As loading continues, the contrast in deformation between coal and rock mass becomes evident, particularly with the contact effect at the coal-rock interface. At this stage, the coal body moves into the plastic stage (*a*_2_*b*_2_), while the rock mass remains in the elastic stage (*a*_1_*b*_1_), and damage starts from the contact surface. With continuous loading, the destruction of the coal body is mostly complete, but the stress doesn’t reach the overall strength of the coal-rock combination structure yet. The rock mass starts to contribute while the stored elastic energy is released. However, the discharged elastic energy does not initiate the destruction of the rock mass. Consequently, the coal body directly loses its load-bearing capacity. This closely resembles the failure mode found in the experimental results of the upper rock and lower coal combination structure, thus verifying the accuracy of said results.

## Numerical simulation of loading on coal-rock composite structures

Based on the PD theory, an axial loading program for coal-rock monomers and different coal-rock composite structures was written in Python. Using an atomic-molecular parallel simulator for computation, the results were visualized and an energy distribution diagram was exported. To make the simulation results more accurate, relaxation time was increased to allow the simulation samples to return to an equilibrium state. During the computation process, the simulator also applies the PD model to simulate the mechanical properties of the samples, introducing the dynamic control equation of the PD theory (Eq 16) into the simulator [27,28].


$$\rho (x)\ddot{u}(x,t)=\int_{Hx}f(x^{\prime },x,u(x,t),u(x,t),t)dV^{\prime }+b(x,t)$$ (16)

Where the material points x and xʹ produce the displacement of $\ddot{u}(x,t)$ and u(xʹ,*t*) respectively at time *t*; The relative position vector of the two particles at the initial time is expressed as xʹ-x = ξ; The relative displacement vector between two points after time t is u(xʹ,*t*)-u(x,*t*) = η; The relative position vector after deformation is ξ+η; *ρ*(x) and $\ddot{u}(x,t)$ represent the density and acceleration of particle x at time *t*, respectively; b(x,*t*) represents the external force on the material point x; Function f is the interaction force function between x and xʹ. In other words, function f is only related to the deformation of the bond between x and xʹ.

Based on the bond-based PD theory, the constitutive force function f is derived from the relationship established by Li et al [29], which is:

$$f(n,\eta )=\{\begin{array}{l} cs(1-D)\frac{\eta +\xi }{\left|\eta +\xi \right|},s_{c}<s<s_{ec}\cup s_{et}<s<s_{t}\\ cs\frac{\eta +\xi }{\left|\eta +\xi \right|},s_{ec}<s<s_{et}\\ 0,\mathrm{other} \end{array}$$ (17)

Where *s*_*et*_ and *s*_*ec*_ are the linear elastic elongation when the bond is stretched and compressed, corresponding to the elastic deformation; c is the elastic stiffness of the key and is a constant, called the ’micro-modulus’, similar to spring stiffness.

The numerical model is a cylindrical specimen with a size of *Φ*50mm×100mm.The proportion of coal and rock is set to 1:1, 1:2, 2:1 respectively. Fix the interface of the composite structure to eliminate the influence of interface slip, so as to achieve a conclusion similar to the experimental results. However, the influence of interfacial dolomite glue in the experiment was ignored in the simulation. After defining the physical and mechanical parameters such as density, elastic modulus, and Poisson’s ratio for coal and rock, the model was discretized into 250,000 material points, with *δ* = 3.015Δ. Finally, apply axial load at the same rate as in the experiments, with the time step set to 20,000 steps [30]. It is worth noting that the simulator removes the volumetric term during calculations, resulting in the calculated "stress" having the unit of energy. Therefore, in order to make the output stress consistent with the actual situation, the ’compute v all voronoi/atom’ and other commands are used for subsequent conversion. After conversion, the maximum stress of damage in the coal is about 40 MPa (1.0e-8).

**Fig 13:**
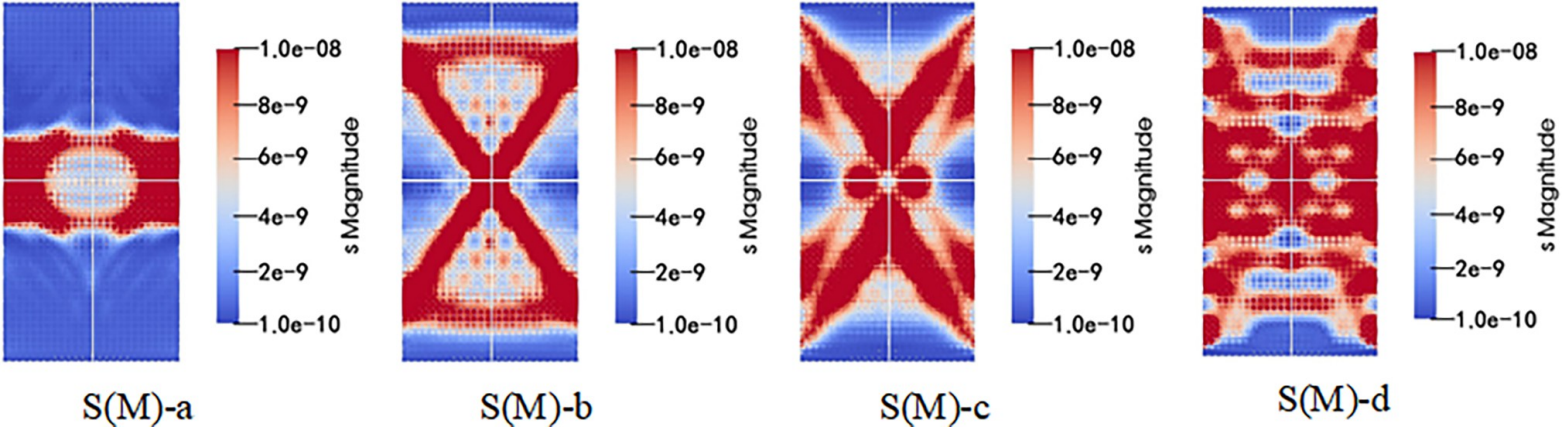
Schematic diagram of stress distribution in whole loading process of single coal or rock.

**Fig 14:**
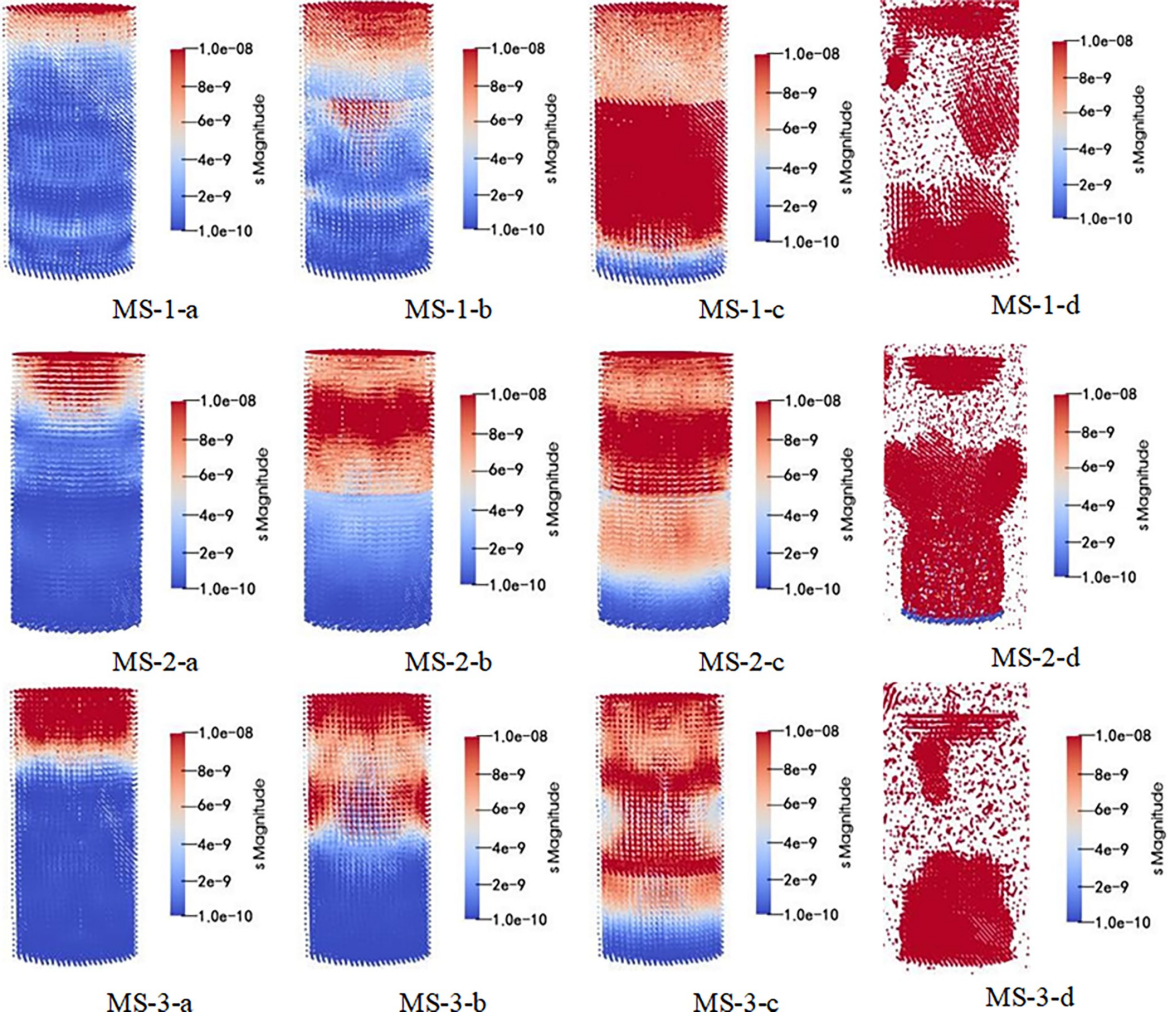
Schematic diagram of stress distribution in whole loading process of upper coal and lower rock composite structure.

**Fig 15:**
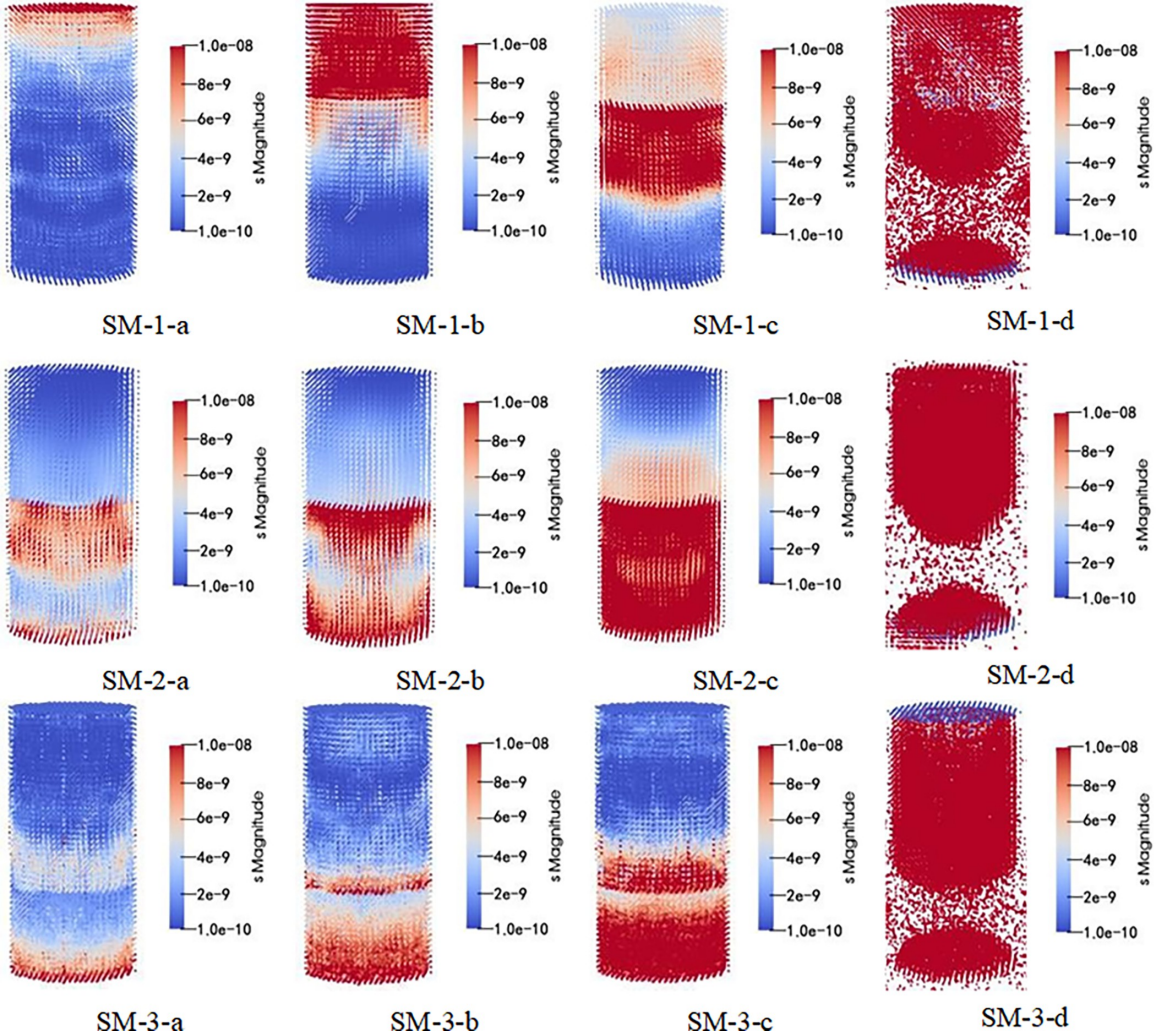
Schematic diagram of stress distribution in whole loading process of upper rock and lower coal composite structure.

After subsequent visualization analysis and processing, simulation results can be obtained (the monomer simulation is the main view, and the combined structure is slightly inclined), and the simulation results are shown in Figs 13–15.

As seen in Figs 13–15. During the whole loading process of the specimen, the energy released by the micro bond fracture is aggregated in an ’ X ’ shape. As the pressure increases, internal energy diffuses, and after reaching the yield stress, the sample exhibits an "X" type failure. Among them, the distribution of coal monomer is more uniform and full of the whole model, while the energy accumulation of the coal composite structure is not obvious, which is mainly in the form of "X" during macro crushing, indicating that the participation of weak components (coal) has a greater impact on the overall model.

**Fig 16:**
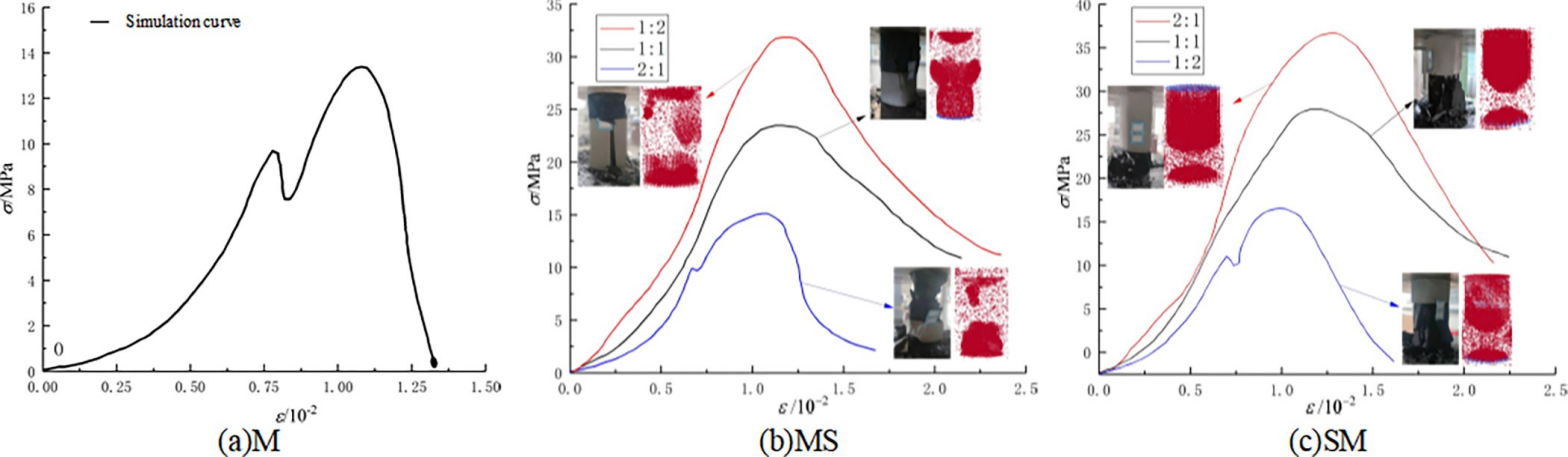
The stress-strain curve of the whole process of specimen loading.

In addition, the combined structure of upper coal and lower rock exhibits overall model fragmentation during macroscopic fragmentation, while the combined structure of upper rock and lower coal exhibits a state of lower coal fragmentation and upper rock mass being relatively intact. This is because as the proportion of coal in the upper coal and lower rock composite increases, the ultimate energy storage of the coal part gradually increases, leading to a more significant "X" type failure phenomenon in the overall model; As the proportion of coal in the upper rock and lower coal composite increases, the ultimate energy storage in the rock mass gradually increases, leading to a more significant "X" type failure failure phenomenon in the model coal. After loading the sample, the stress-strain curve are shown in Fig 16.

As show by Fig 16, the compressive strength of the coal-rock composite structure with different proportions decreases with the increase of weak components, indicating that the internal stress conduction rate of the model is directly proportional to the compressive strength. Moreover, the compressive strength of the upper rock and lower coal composite structure is higher than that of the corresponding upper coal and lower rock composite structure in different proportions, indicating that the upper rock and lower coal composite structure are more compressive, which means they are more prone to rockburst. The simulation results are closer to reality. Calculate the *W*_ET_ and *W*_CF_ of the model, as shown in Table 6.

**Table 6:** Calculation results of *W*_ET_ and *W*_CF_ for simulated samples.

Name	M	MS-1	MS-2	MS-3	SM-1	SM-2	SM-3
***W***_**ET**_	6.40	10.21	6.00	4.37	6.24	6.79	12.44
***W***_**CF**_	3.90	4.54	2.41	1.29	1.94	3.19	6.02

The various indices of the simulated sample are lower than those of the experiment, and the slope of the fitting curve is smaller. This is because the established model is a uniform model without external interference, while the uniformity of the model used in the experiment is poor, and there are factors such as time effect and operation error, which produce certain errors and can be ignored. According to the impact tendency index of each index, the impact tendency of the simulated sample is determined, as shown in Table 7.

**Table 7:** The tendency of simulated sample rockburst occurrence.

Name	*W*_ET_	*W*_CF_
**M**	Strength	Strength
**MS-1**	Strength	Strength
**MS-2**	Strength	Feebleness
**MS-3**	Feebleness	Nil
**SM-1**	Feebleness	Feebleness
**SM-2**	Strength	Strength
**SM-3**	Strength	Strength

The impact tendency of the simulated sample is the same as that of the corresponding sample in the experiment, and there is also a good correspondence with the experimental results, indicating that the experimental results are true and effective. Therefore, the lithology, composite state, and composite ratio of coal rock composite structure are the main reasons that affect the occurrence of rockburst. The experimental and simulation results can provide reference for predicting the tendency of rockburst occurrence in practical engineering.

## Conclusion

In this paper, the variation of mechanical properties of coal-rock composite structure with different combination states and different composite ratios during loading was studied. The rock elastic deformation energy index (*W*_ET_) and rock impact energy index (*W*_CF_) were combined to determine the impact tendency of different coal-rock composite structure. And based on peridynamics, load simulation was conducted on the coal-rock composite structure using Lammps software, and the internal energy changes were analyzed to verify the experiment. This study can provide theoretical support for underground engineering construction, such as judging the tendency of rock mass shedding or impact around the roadway in roadway support, prevent casualties, and reduce equipment damage and costs. The results show that:

(1) The compressive strength, elastic energy and impact energy of coal-rock composite structure are between coal monomer and rock monomer, and are more inclined towards weak components, namely the proportion of coal. The higher the proportion of coal, the lower the mechanical parameters.

(2) According to the judgment results of the two energy indexes of rock elastic deformation energy index (*W*_ET_) and rock impact energy index (*W*_CF_), the impact tendency of the roof is stronger than that of the floor, and the impact tendency of the SM composite structure with coal-rock ratio of 1:2 is the strongest among different proportion composite structure.

(3) In the whole process of simulated sample loading, the energy released by bond fracture at the micro level is ’ X ’-type aggregation, and finally ’ X ’-type failure. And with the participation of weak components (coal), the impact on the overall model’s fragmentation is significant, which is consistent with the experimental results, indicating that the experimental results are true and effective. The different composite states and ratios of roof and coal, coal and floor are important factors for the damage of coal-rock composite structure, which has reference significance for predicting the occurrence of rockburst.

The research results indicate that coal-rock composite structures in underground tunnels are also prone to impact ground pressure incidents, with a stronger impact tendency when the load-bearing surface is rock, especially when the coal-to-rock ratio is 1:2. However, due to the limitations in experimental workload, this study only examined three ratios—1:1, 1:2, and 2:1. Further studies should continue to identify the coal-to-rock ratio with the highest impact tendency to effectively predict the occurrence of impact ground pressure in coal-rock composites. These uncertainties present an intriguing topic for further investigation.

## Supporting information

S1 File Original PYTHON code for simulating the load of coal-rock composite structure (different models need to be adjusted). (DOCX)

## References

[1] Miao L, Niu Y, Shi B. Impact Dynamic Test Study of Rock-Coal-Rock composites at Different Strain Rates. Vibration and Shock. 2019; 38:137–143. 10.13465/j.cnki.jvs.2019.17.018.

[2] Duan W. Occurrence mechanism and prevention technology of rock burst in coal seam mining under deep well with thick hard roof. Shanxi Coking Coal Science and Technology. 2021; 45:16–19.(in Chinese).

[3] Du T, Li K, Lan H, Liu X. Analysis of the disaster process caused by rock burst in near-upright extra-thick coal seams. Journal of Mining and Safety Engineering. 2018; 35:140–145. 10.13545/j.cnki.jmse. 2018.01.020.

[4] Cao J, Dou L, Konietzky H, Zhou K, Zhang M. Failure mechanism and control of the coal bursts triggered by mining-induced seismicity: a case study. Environmental Earth Sciences. 2023;82(7). 10.1007/S12665-023-10856-9.

[5] Wang C. Monitoring and warning technology of acoustic emission in static loading rock burst. Coal Science and Technology. 2021; 49(06): 94–101. 10.13199/j.cnki.cst.2021.06.011.

[6] Liu J, Zheng Z, Zhou H, Zhou N, Wang Y, Sun M. Mechanical characteristic of similar weakly cemented soft rock under directional shear stress path and modified Lade-Duncan criterion. International Journal of Geomechanics. 2024; 51(11): 04024260-1-12. 10.1061/IJGNAI.GMENG-10680.

[7] Ma Q, Liu X, Tan Y, Wang R, Xie W, Wang E, et al. Experimental study of loading system stiffness effects on mechanical characteristics and kinetic energy calculation of coal specimens. Rock Mechanics and Rock Engineering. 2024; 1–17. 10.1007/s00603-024-04054-7.

[8] Li C, Xu Y, Zhang Y, Li H. Study on energy evolution and fractal characteristics of cracked coal-rock-like combined body under impact loading. Chinese Journal of Rock Mechanics and Engineering. 2019; 38(11): 2231–2241. 10.13722/j.cnki.jrme.2019.0446.

[9] Li C, Xu Y, Ye Z. Energy dissipation and crushing characteristics of coal-rock-like combined body under impact loading. Chinese Journal of Geotechnical Engineering. 2020; 42(5): 981–988.

[10] Liu X, Tan Y, Ning J, Lu Y, Gu Q. Mechanical properties and damage constitutive model of coal in coal-rock combined body. International Journal of Rock Mechanics and Mining Sciences. 2018; 110140–150. 10.1016/j.ijrmms.2018.07.020.

[11] Zuo J, Song H, Chen Y, Li Y. Post-peak progressive failure characteristics and nonlinear model of coal-rock combined body. Journal of China Coal Society. 2018; 43(12): 3265–3272. 10.13225/j.cnki.jccs.2018.0292.

[12] Zuo J, Chen Y, Cui F. Investigation on mechanical properties and rock burst tendency of different coal-rock combined bodies. Journal of China University of Mining & Technology. 2018; 47(1): 81–87. 10.13247/j.cnki.jcumt.000795.

[13] Zuo J, Chen Y. Investigation on crack recovery effect of coal-rock combined body under the influence of unloading. Journal of China Coal Society. 2017; 42(12): 3142–3148. 10.13225/j.cnki.jccs.2017.0682.

[14] Zhao Y, Ling C, Liu B, He X. Fracture evolution and energy dissipation of overlying strata in shallow-buried underground mining. Journal of Mining and Safety Engineering. 2021; 38(01): 9–18+30. 10.13545/j.cnki.jmse.2020.0212.

[15] Shu J, Li H, Huang X, Zhang J. Limiting dynamics of stochastic heat equations with memory on thin domains. Applicable Analysis. 2023; 102(15): 4092–4113. 10.1080/00036811.2022.2103796.

[16] Gfrerer M. Rigorous code verification for non-linear Kirchhoff-Love shells based on tangential differential calculus with application to Isogeometric Analysis. Finite Elements in Analysis & Design. 2023; 227. 10.1016/J.FINEL.2023.104041.

[17] Li S, Lu H, Huang X, Qin R, Mao J. Sensitivity analysis of notch shape on brittle failure by using uni-bond dual-parameter peridynamics. Engineering Fracture Mechanics. 2023; 291. 10.1016/J.ENGFRACMECH.2023.109566.

[18] Liu Q, Yu Y, Hu Y, Madenci E. Thermomechanical modeling of pellet-cladding interaction using state-based peridynamics. Engineering Fracture Mechanics. 2023; 290. 10.1016/J.ENGFRACMECH.2023.109496.

[19] China Coal Research Institute. GB/T 23561.7−2009Methods for determining the physical and mechanical properties of coal and rock—Part 7: methods for determining the uniaxial compressive strength and counting softening coefficient. Beijing: China Standards Press, 2009. (in Chinese).

[20] Chen G, Li T, Yang L, Zhang G, Li J, Dong H Mechanical properties and destruction mechanism of different coal rock ratio and combination methods. Mining and Layer Control Engineering Journal. 2021; 3(02): 84–94. 10.13532/j.jmsce.cn10-1638/td.20210108.001.

[21] Feng G, Yu Z, Zhang Q. Study on the top coal crushing mechanism of comprehensive mining and discharging top coal mining. Journal of Fuxin Institute of Mining and Technology (Natural Science Edition). 1992; 11(3):51–54. (in Chinese).

[22] Zuo J, Xie H, Meng B, Liu J. Experimental research on loading-unloading behavior of coal-rock combination bodies at different stress levels. Rock and Soil Mechanics. 2011; 32(5):1287–1296. 10.16285/j.rsm.2011.05.028.

[23] Kidybinski A. Bursting liability indices of coal. Interna-tional Journal of Rock Mechanics and Mining Sciences&Geomechanics Abstracts. 1981; 18(4):295–304. 10.1016/0148-9062(81)91194-3.

[24] MT/T 174–2000, the measurement method of coal seam impact tendency classification and index. 2000. (in Chinese).

[25] Benilov E. Does Maxwell’s hypothesis of air saturation near the surface of evaporating liquid hold at all spatial scales?. Journal of Fluid Mechanics. 2023; 971(420): A20–A20. 10.1017/JFM.2023.667.

[26] Nguyen T, Li J, Sun L, Tran D, Xuan F. Viscoelasticity Modeling of Dielectric Elastomers by Kelvin Voigt-Generalized Maxwell Model. Polymers. 2021; 13(13): 2203–2203. doi: 10.3390/polym13132203 34279347 PMC8272131

[27] Huang D, Zhang Q, Qiao P, Shen F. Peridynamics Methods and Their Applications. Advances in Mechanics. 2010; 40(04): 448–459.

[28] Liu N, Hu M, Zhou F. Impact failure of single-crack circular orifice plate based on bond-base peridynamic theory. Engineering Mechanics. 2020; 37: 9–17.

[29] Li H, Liu J, Wang L, Ren T. Study on mechanical properties and energy change of rock materials in whole splitting process based on peridynamics. Sci Rep. 2023; 13: 3221. doi: 10.1038/s41598-023-30394-5 36828861 PMC9958110

[30] Wang Y, Liu X, Chen D, Wang D, Yang Y, Chen D. Research on damage characteristics of gas-bearing outburst coal under different loading rates under loading. Coal Mine Safety. 2020; 51: 25–30. 10.13347/j.cnki.mkaq.

