# Supplementary material for: Investigation into the mechanical properties and impact tendency of coal-rock composite structures with peridynamics: A study on predicting the occurrence tendency of dynamic pressure in coal-rock structures
Source: PLoS One. 2024 Dec 4;19(12):e0314927. doi: 10.1371/journal.pone.0314927 (PMC11616808; doi:10.1371/journal.pone.0314927)
Supplement: S1 File — (DOCX) [file pone.0314927.s001.docx]

units si

dimension 3

boundary s s s

atom_style peri

atom_modify map array

neighbor 0.001 bin

neigh_modify delay 5000

lattice sc 0.0005

region box block -0.5 0.5 -0.5 0.5 -0.5 0.5 units box

region target1 cylinder y 0.0 0.0 0.005 -0.01 0 units box

region target2 cylinder y 0.0 0.0 0.005 0 0.01 units box

create_box 2 box

create_atoms 1 region target1

create_atoms 2 region target2

write_data zh_model.data

region top cylinder y 0.0 0.0 0.005 0.010 INF units box

region bottom cylinder y 0.0 0.0 0.005 INF -0.010 units box

#group target region target

group top region top

group bottom region bottom

group target1 region target1

group target2 region target2

group realtarget subtract all top bottom

set group all volume 1.25e-10

set group target1 density 1500

set group target2 density 2200

pair_style peri/pmb

pair_coeff * * 2.8611e21 0.0015001 0.0186 1e-7

run 3000

#minimize 1.0e-2 1.0e-2 10000 10000

fix top_rigid top rigid single

fix bottom_rigid bottom rigid single

fix noforce_bottom bottom setforce 0.0 0.0 0.0

fix noforce_top top setforce 0.0 0.0 0.0

velocity bottom set 0.0 0.0 0.0 units box

velocity top set 0.0 0.0 0.0 units box

velocity top set 0.0 -0.8 0.0 units box

#velocity all set 0.0 0.0 0.0 sum no units box

#fix top_move top move linear 0 -50 0

fix wall_bottom all wall/reflect ylo -0.011 units box

variable y equal "0.011 - step*dt*0.09"

fix wall_top all wall/reflect yhi v_y units box

#fix nve_realtarget realtarget nve

#fix 1 all nve/limit 0.1

fix 1 all nve

compute C1 all damage/atom

compute s all stress/atom NULL

compute p all pe/atom

compute k all ke/atom

thermo 200

thermo_style custom step temp enthalpy etotal pe ke press vol density lx ly lz elapsed

dump D1 all custom 200 dump3.peri id type x y z c_p c_k c_C1 c_s[1] c_s[2] c_s[3]

timestep 2e-7

run 10000
